# Split Inferior Pedicle: The 1-Stage Augmentation Mastopexy for Grade 3 Ptosis

**DOI:** 10.1093/asjof/ojac017

**Published:** 2022-03-14

**Authors:** Alexia Stamatiou, Christina Stamatiou, Vassilis Stamatiou

**Affiliations:** 1 Department of General Surgery, Weill-Cornell NYP, New York City, NY, USA; 2 Charles E. Schmidt College of Medicine, Florida Atlantic University, Boca Raton, FL, USA

## Abstract

In this article, the authors describe the 1-stage surgical technique that has been used by the senior author since 1990 for all his primary grade 2 and grade 3 augmentation-mastopexies. The article provides a safe, simple, and reproducible approach to a challenging procedure, one that tends to be the most litigious in aesthetic breast surgery. The key points of this technique are simple preoperative markings; the augmentation is performed with a true submuscular placement of smooth implants; and the mastopexy is performed with an inferior pedicle technique with unlimited skin flap undermining and no limitation of nipple elevation. Seventy-eight consecutive cases of augmentation mastopexy performed since 1999 by a single surgeon (V.S.) using the same technique were reviewed. The age range was 26-62 years old; the range of implant volume was 150-375 cc; and the follow-up time period was from 1 year to 22 years. After an extensive patient case review, the authors enforce true extended submuscular augmentation (TESMA) as a reliable augmentation technique that can be used as the first procedure in any grades 2 and 3 ptosis, 1-stage augmentation-mastopexies including bariatric cases. The authors believe that the split inferior pedicle for the mastopexy in combination with the TESMA is a breakthrough approach that eliminates the second stage for any augmentation mastopexy procedure. No major complications such as nipple necrosis, implant bottoming, malpositioning, or extrusions were observed. It is a safe, simple, and reproducible procedure.

**Level of Evidence: 4:**

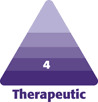

Since first described by Gonzalez-Ulloa^1^ and Regnault,^2^ single-stage augmentation mastopexy remains a technically challenging procedure. In our view, the augmentation-mastopexy is a procedure that has been plagued by the notion of the opposing forces resulting from the combination of the 2 procedures.^3-11^ The problem originates from the persistent use of the dual plane,^12^ subglandular, and subfascial^13^ augmentation as part of the procedure which is then combined most often with a superior pedicle flap for the mastopexy.^14-16^ When these types of augmentation are used, the implant lacks muscle support at the inferior pole, and, therefore, the “gravity effect” of the implant opposes the intended mastopexy lifting effect. This process creates the “opposing forces” of the augmentation and the mastopexy, which contributes to results that are not long-lasting, early postoperative complications, reoperations, and technically difficult problems to solve. These problems can be circumvented by preserving true muscular coverage at the inferior pole. By supporting the implants with a true submuscular pocket at the inferior pole, the mastopexy is performed using an inferior pedicle that allows wide undermining without any vascular compromise and excellent parenchymal redistribution. The inferior pedicle in combination with the muscular closure of the augmentation gives additional tissue coverage for the implant at the inferior pole eliminating the possibility of the implant extrusion and development of a low double bubble (LDB).

The “split inferior pedicle” technique was developed by the senior author (V.S.) in 1990. The technique consists of a combination of a true extended submuscular augmentation (TESMA) followed by an inferior pedicle mastopexy. The pedicle is split transversely by the augmentation incision and, therefore, is referred to as a “split inferior pedicle.”

The senior surgeon originally performed submusculofascial augmentations as described by Bostwick,^17^ for all his primary augmentations but then progressed to developing an inferiorly based muscle cuff for full muscle coverage which he refers to as a TESMA. This technique is done strictly only through the inframammary fold (IMF) approach.^18^ It allows a wide range of implant sizes to be used and provides long-lasting natural results in all his primary augmentations. With an unwavering commitment to the TESMA technique, and striving to perform a safe and long-lasting 1-stage augmentation-mastopexies, he developed the “split inferior pedicle” technique. For over 30 years, the “split inferior pedicle” augmentation mastopexy has been proven to be safe, reliable, and reproducible with long-lasting results without experiencing any major complications such as nipple-areola complex (NAC) necrosis, implant bottoming, malpositioning, or extrusions.^4-11^

## METHODS

Since 1999, seventy-eight consecutive cases of primary 1-stage augmentation-mastopexies for all grades 2 and 3 breast ptosis have been performed in Greece using the TESMA augmentation and the split inferior pedicle mastopexy. For all of these procedures, patients provided preoperative informed consent and also photograph consent, and the principles of the declaration of Helsinki^19^ regarding the ethical treatment of human patients in research were followed. The only exclusion criterion for the mastopexy is the history of any previous breast surgery including biopsies. The follow-up range of the study is 1 to 22 years, and the age range was 26 to 62 years old (Table 1). Since 2006, we incorporated, invariably in all the cases, the plication of the inferior pedicle as described by JP Rubin.^20-22^ Since then, there has not been any deviation or variation of this technique.

**Table 1.** Implant Size and Follow-up Range

Variables	Data
Follow-up range	1-22 years
Follow-up mean	8.2 years
Implant size range	150-375 cc
Implant size mean	270 cc
Age range	26-62 years
Age mean	36.3 years

Ranges and means of the variables for each patient. These variables include the years of postoperative follow-up, the implant sizes used, and the patient ages.

The size of the implants is determined intraoperatively using sizers taking into consideration the wishes of the patient for the size of the breast and the body type. We elected to only use Mentor (Santa Barbara, CA) smooth round moderate and moderate plus silicone implants. The size of the implant is limited only by the need for complete muscle coverage and in our practice ranges from 150 to 375 cc (μ = 270 cc).

## Steps of the Procedure

### Preoperative Markings

**Figure 1.**
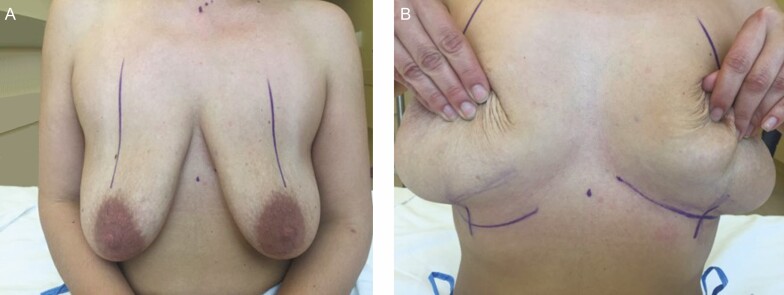
Preoperative markings on a 38-year-old female with grade 3 ptosis: (A) markings centered on the midclavicular lines and (B) markings for the projected inframammary folds.

The preoperative markings (Figure 1) consist of the midclavicular lines extending to the IMFs and the augmentation incisions at the IMF. The augmentation incision markings of 4 cm are centered at the midclavicular lines on the IMF. In the case of uneven IMFs, the higher IMF is lowered to the level of the contralateral IMF. We may lower both incisions to a new projected IMF. The new projected IMF is determined preoperatively by displacing the breast tissue inferiorly mimicking the effect of the implants and mastopexy to the inferior pole.

### True Extended Submuscular Augmentation

**Figure 2.**
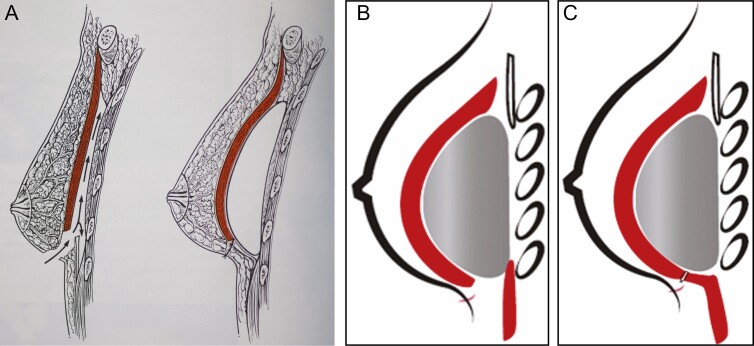
Three techniques for breast augmentation: (A) the Submusculofascial technique, (B) the type 1 dual plane approach, and (C) true extended submuscular augmentation (TESMA). The illustrations are courtesy of: Aesthetic and Reconstructive Breast Surgery, John Bostwick III, pg 104, Copyright Elsevier (1983) ISBN 0-016-0731-0.

**Figure 3.**
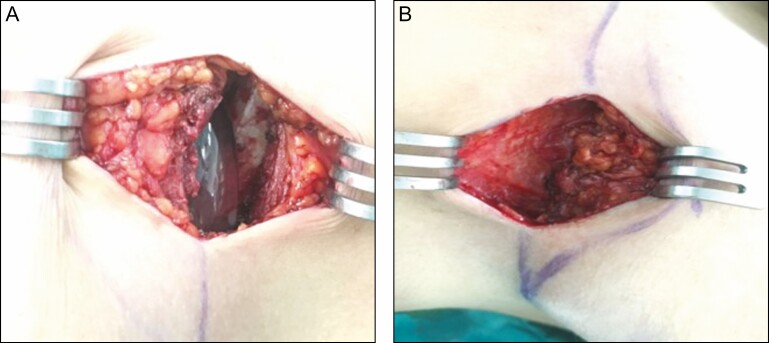
(A) The submuscular placement of the implant in a 38-year-old female with the creation of the inferiorly based muscular cuff; (B) the direct approximation of the insertion of the pectoralis major muscle to the inferiorly based cuff, resulting in complete muscular coverage of the implant.

There are 3 variations of submuscular breast augmentation: the submusculofascial, dual plane, and TESMA. The submusculofascial and dual plane techniques can be done either by IMF or by periareolar access (Figure 2). The TESMA, however, can only be done through the IMF approach. The insertion of the pectoralis major muscle is transected, and the muscle is elevated from the ribs and the pectoralis minor. Medially, the muscle is detached from the third to the seventh ribs using cautery. Laterally, the dissection is mostly blunt to the limits of the pocket, especially over the fourth and fifth intercostal neurovascular bundles, protecting the sensory nerve to the NAC. An inferiorly based cuff is then raised opposite to the pectoralis major transected edge, by elevating the insertion of the abdominal wall muscles, primarily the rectus abdominis. Guided by the use of sizers, we deepen the cuff to the new IMF as needed. The smooth silicone implants are then inserted, and the edge of the pectoralis major is sutured meticulously to the edge of the muscle cuff with interrupted 3-0 vicryl sutures (Figure 3). In the process of suturing the pectoralis major to the muscle cuff, we eliminate the gap between the insertion of the pectoralis major and the insertion of the rectus abdominis muscle, thereby forming an internal muscular bra. Lowering the IMF and extending the submuscular pocket inferiorly allow the placement of larger size implants with complete muscle coverage without having the limitation of the higher placement of smaller implants as it was described by Dempsey and Latham’s^23^ original true submuscular technique.

### Post-augmentation Wise Pattern Markings

In the Wise pattern, the augmentation incisions correspond transversely at the base of the inferior pedicle, therefore creating a “split inferior pedicle.”

**Figure 4.**
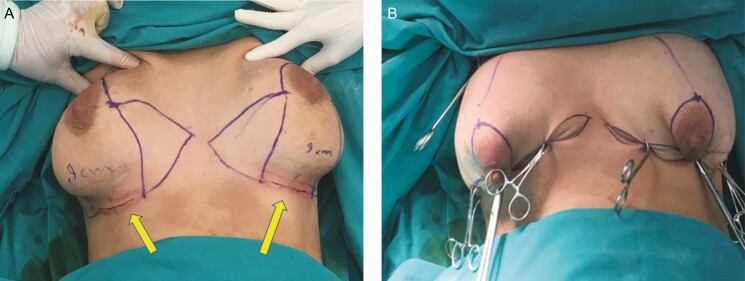
(A) The Wise pattern that was drawn with the implants in place and the 38-year-old female patient in the upright position after the closure of the augmentation incisions (yellow arrows) to the subcutaneous level. (B) A mock closure of the drawn Wise pattern with the implants in place for confirmation of the markings.

With the patient in the upright position, the implants in place, and the augmentation incision closed to the subcutaneous level, the Wise pattern is drawn with 7 cm vertical limbs (Figure 4). The NACs are marked at the end of the procedure. The Wise pattern markings are confirmed by placing towel clips in order to evaluate the degree of cleavage, shape and size of the breast, and tightness of T-closure that will be produced at the end of the procedure, while the muscle relaxant is still in effect.

#### “Split Inferior Pedicle” Mastopexy

**Figure 5.**
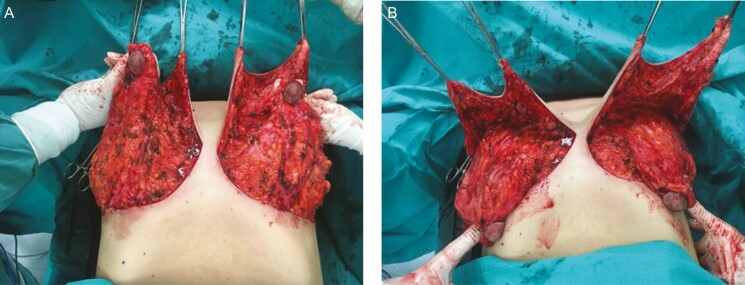
The development of the inferior pedicle in a 38-year-old female with wide undermining of the superior pole and the implants in place. (A) Pedicles are positioned cephalad and (B) pedicles are positioned caudally.

**Figure 6.**
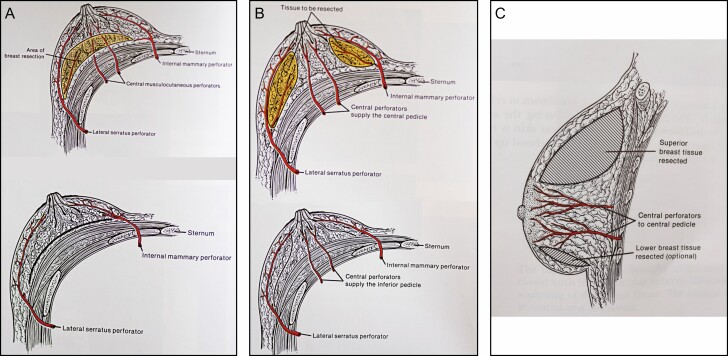
The vascular supply of the superior and inferior pedicles, respectively, before and after the excision or undermining in the area of the yellow-shaded breast tissue: (A) with the superior pedicle, the central musculocutaneous perforators to the nipple-areola complex (NAC) are sacrificed; (B) with the inferior pedicle, the central musculocutaneous perforators to the NAC are preserved; (C) a lateral view of the inferior pedicle showing preservation of the blood supply to the NAC after potential breast tissue excision in the shaded areas. The illustrations are courtesy of: Aesthetic and Reconstructive Breast Surgery, John Bostwick III, pgs 172 (A), 186 (B), 187 (C), Copyright Elsevier (1983) ISBN 0-016-0731-0.

The procedure to be performed is an inferior pedicle mastopexy with unlimited undermining of flaps and elevation of the NAC as it is typically done with a breast reduction (Figure 5). We elect to de-epithelialize the flap for the mastopexy in order to facilitate the plication of the inferior pedicle that will be described below. The critical issue at this point is the preservation of the blood supply to the pedicle which is split by the preceding augmentation. To achieve that, the surgeon must secure the blood supply to the inferior pedicle. The breast tissue that is attached to the underlying pectoralis major needs to remain undisturbed in order to preserve the musculocutaneous perforators that supply the central pedicle and the NAC (Figure 6). In order to keep the blood supply to the inferior pedicle intact, dual plane, subfascial, subglandular placement of implants and periareolar approach are prohibited. The TESMA augmentation that is done only through the IMF approach allows the development of the inferior pedicle without vascular compromise to the pedicle and the NAC. The resection of skin/breast tissue, laterally and medially to the inferior pedicle, is performed 1-2 cm above the fascia in order to preserve additional blood supply to the pedicle. We then complete the development of the inferior pedicle by freely undermining the skin flaps and shaving any excess tissue needed at the superior pole. After the plication of the inferior pedicle, as described below, the vertical skin incisions are marked and closed at 4.5 cm length. The NACs are usually marked with 45 mm diameter and placed in 42 mm diameter corresponding openings. Drains are not used.

## Vascular Anatomy

### Plication of the Inferior Pedicle

**Figure 7.**
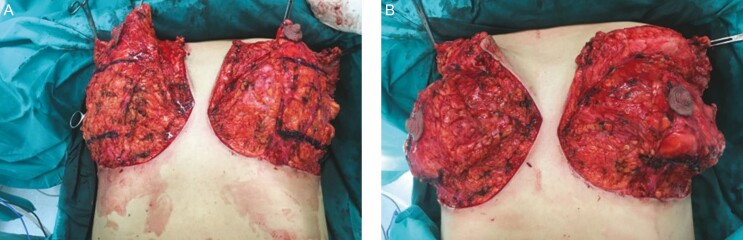
(A) The markings on the inferior pedicle before plication and (B) the shortened inferior pedicle after the plication in a 38-year-old female.

The de-epithelialized inferior pedicle is then plicated to a total vertical length of 6 cm marking horizontally 3 cm from the areola and 3 cm from the IMF (Figure 7). The plication does not compromise the blood supply to the pedicle as there are no vessels originating from the inferior base of the pedicle. The inferior pedicle is de-epithelialized, not to preserve blood supply but merely to envelope the inferior pole and make the plication sutures of the pedicle safer. The advantage of the plication is 2-fold. Firstly, it helps lessen the possibility of long-term breast tissue bottoming at the inferior pole as is seen with inferior pedicle breast reductions. Secondly, it helps eliminate any breast asymmetry by equalizing the length of the pedicles. The plication sometimes creates bulging at the superior pole which can be shaved accordingly for symmetry purposes.

**Figure 8.**
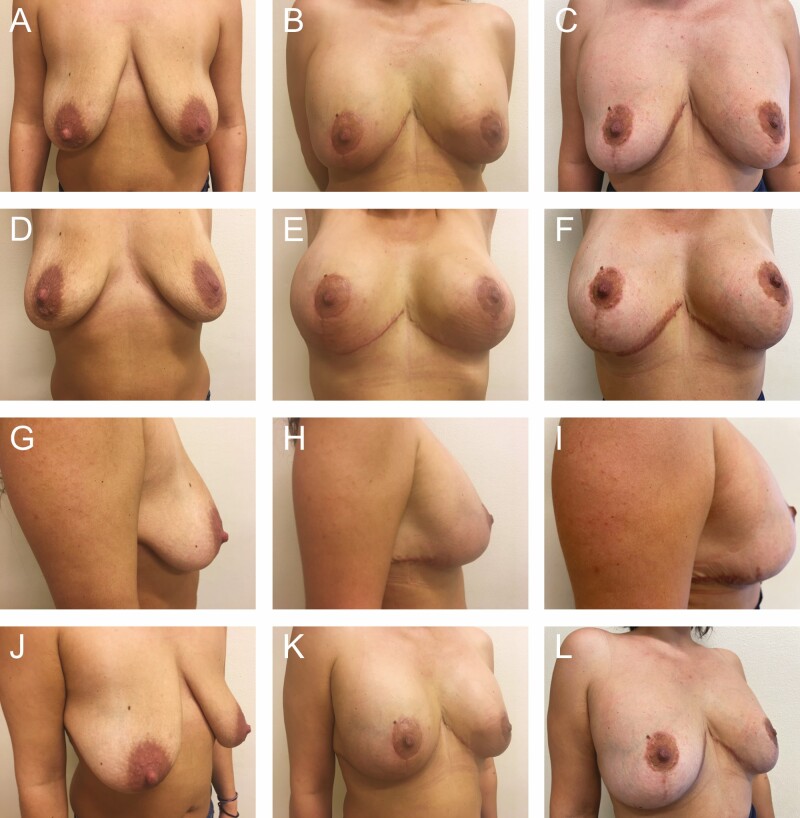
Preoperative photographs of a 38-year-old female with 300 cc implants from various views: (A) frontal, (D) frontal with raised arms, (G) right lateral, and (J) right oblique; postoperative photographs at 3 months: (B) frontal, (E) frontal with raised arms, (H) right lateral, and (K) right oblique; and postoperative photographs at 2 years: (C) frontal, (F) frontal with raised arms, (I) right lateral, and (L) right oblique. Figures 1, 3, 4, 5, and 7 are the intraoperative photos from this same patient.

## RESULTS

**Figure 9.**
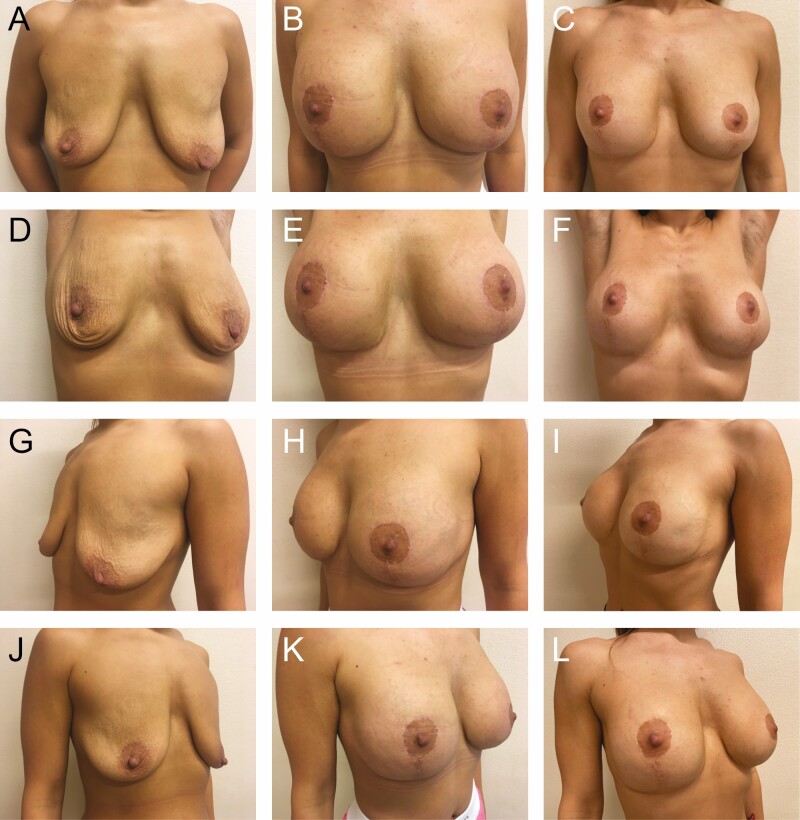
Preoperative photographs of a 30-year-old female with 325 cc implants from various views: (A) frontal, (D) frontal with raised arms, (G) left oblique, and (J) right oblique; postoperative photographs at 3 months: (B) frontal, (E) frontal with raised arms, (H) left oblique, and (K) right oblique; and postoperative photographs at 13 months: (C) frontal, (F) frontal with raised arms, (I) left oblique, and (L) right oblique.

**Figure 10.**
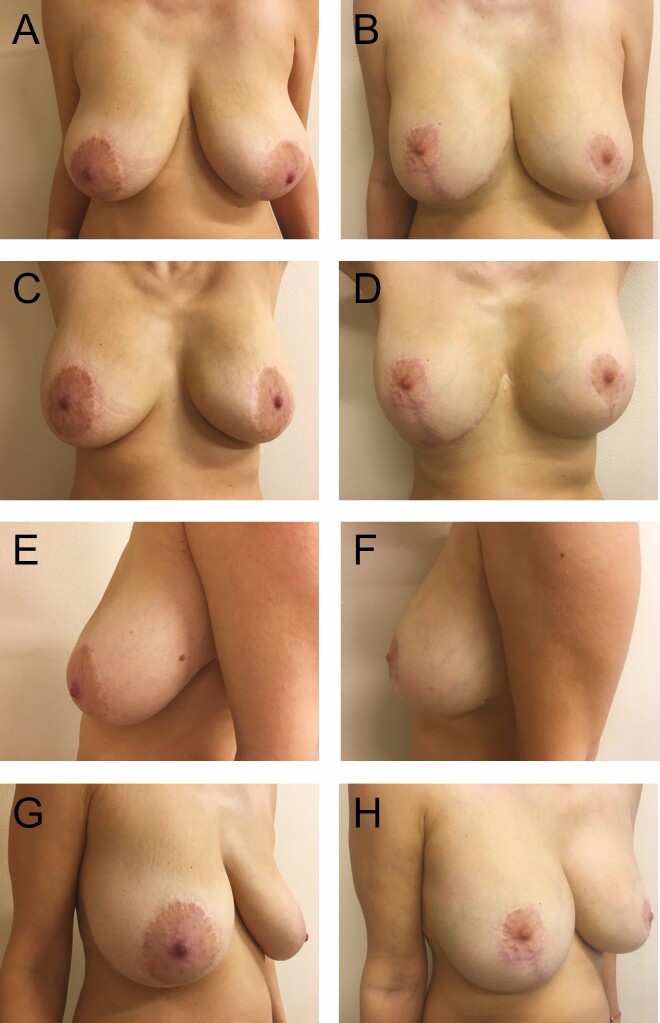
Preoperative photographs of a 45-year-old female with 225 cc implants from various views: (A) frontal, (C) frontal with raised arms, (E) left lateral, and (G) right oblique; postoperative photographs at 2 years: (B) frontal, (D) frontal with raised arms, (F) left lateral, and (H) right oblique.

**Figure 11.**
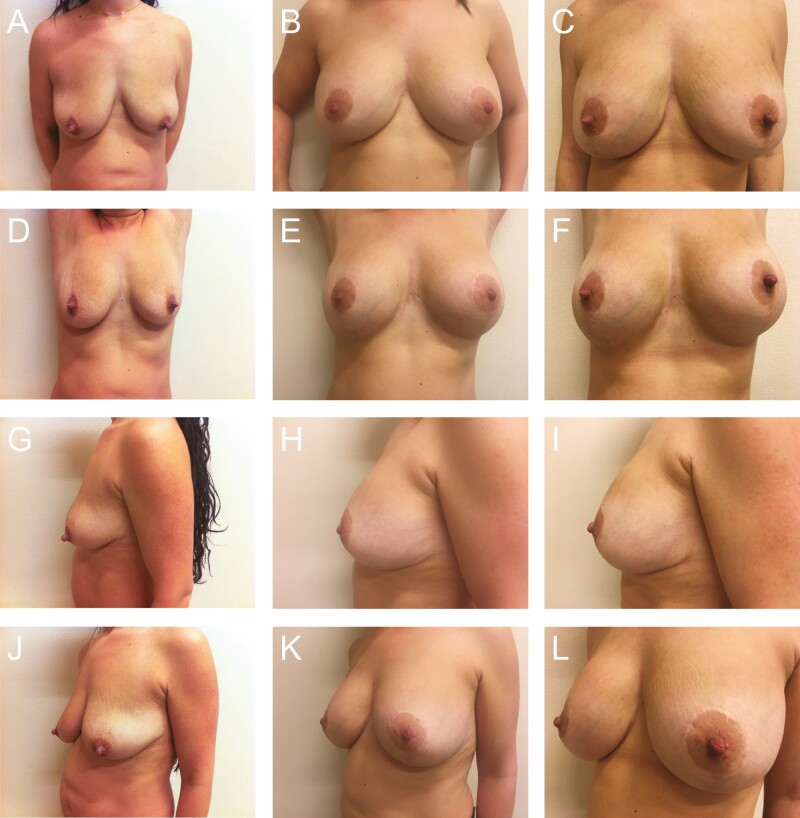
Preoperative photographs of a 33-year-old female with 300 cc implants from various views: (A) frontal, (D) frontal with raised arms, (G) left lateral, and (J) left oblique; postoperative photographs at 2.5 years: (B) frontal, (E) frontal with raised arms, (H) left lateral, and (K) left oblique; and postoperative photographs at 4 years: (C) frontal, (F) frontal with raised arms, (I) left lateral, and (L) left oblique.

**Figure 12.**
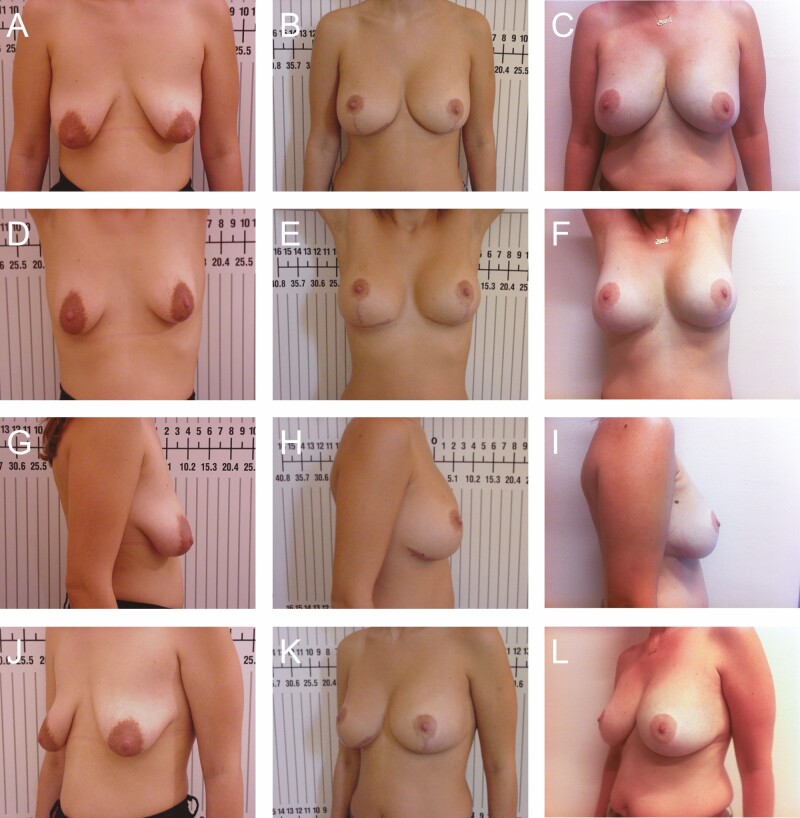
Preoperative photographs of a 30-year-old female with 325 cc implants from various views: (A) frontal, (D) frontal with raised arms, (G) right lateral, and (J) left oblique; postoperative photographs at 1 year: (B) frontal, (E) frontal with raised arms, (H) right lateral, and (K) left oblique; and postoperative photographs at 19 years: (C) frontal, (F) frontal with raised arms, (I) right lateral, and (L) left oblique.

**Figure 13.**
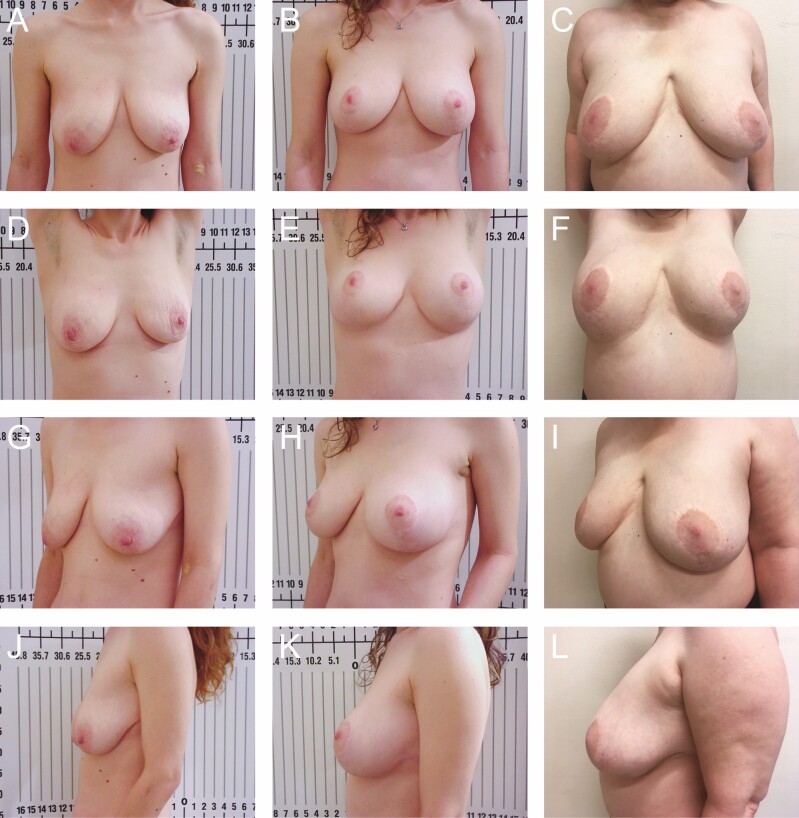
This 37-year-old female patient with 375 cc implants had additional excision of breast tissue from the lateral wedge for contouring purposes. In this series of photographs, the patient was 120 lbs at the time of surgery and 210 lbs at the 19-year follow-up. Preoperative photographs from various views: (A) frontal, (D) frontal with raised arms, (G) left lateral, and (J) left oblique; postoperative photographs at 15 months: (B) frontal, (E) frontal with raised arms, (H) left lateral, and (D) left oblique; postoperative photographs at 19 years: (C) frontal, (F) frontal with raised arms, (I) left lateral, and (L) left oblique.

A total of 78 patients were reviewed. From 1990 to 1999, another 52 patients were lost to follow-up due to the relocation of our practice from Florida to Greece and were not included in the study (patient 1, postoperative photographs: Figure 8; patients 2-6, postoperative photographs: Figures 9-13; patient 7, Videos 1-5).

### Major Complications

Through the follow-up periods, no major complications were encountered, including infections, implant extrusion, nipple-areola or flap necrosis, and malpositioning of implants. Additionally, no reoperations have been required.

### Minor Complications

Complications that occurred include 3 augmentation hematomas (3.84%), 1 mastopexy hematoma (1.28%), 4 superficial “T” point sloughings (5.13%), 6 marginal areoral sloughing (7.69%), and 14 minor scar revisions under local anesthesia (17.95%).

## Discussion

The procedure described is an inferior pedicle mastopexy with unlimited undermining of flaps and elevation of the NAC with the use of smooth round implants of any size that can be covered completely by muscle. The procedure is versatile and is used for all primary grades 2 and 3 ptosis.

As we discuss the procedure, a reference to the augmentation needs to be further analyzed. The TESMA augmentation should not be done by the periareolar approach for 2 reasons. Firstly, by using the periareolar approach and dissecting through the breast tissue, the viability of the flap is compromised. However, by using the IMF approach, the surgeon is able to bypass the breast tissue during the augmentation; therefore, the attachment of the breast to the pectoralis major muscle and the perforators to the NAC remain undisturbed. Secondly, with the IMF access, the inferiorly based muscle cuff is easily developed to provide complete muscle coverage at the inferior pole.

The complete muscle coverage is achieved by suturing the inferiorly based muscle cuff to the opposing edge of the transected pectoralis major. By doing so, the inferior muscle cuff is stretched and overlaps the gap between the insertion of the pectoralis major and the insertion of the rectus abdominis, subsequently creating a true submuscular pocket. This technique counteracts the opposing forces of the augmentation and mastopexy procedures. Therefore, the use of the periareolar approach, dual plane, subfascial, or subglandular augmentation is not compatible with this technique.

The inferior muscle cuff does not allow the development of an LDB. LDB can only be an iatrogenic result of the uneven deepening of the inferior cuff. If an LDB develops and needs to be corrected, the lower aspect of the pocket always has muscular coverage of the implant.

The advantages of using this technique for 1-stage augmentation mastopexy can be divided into 3 parts: technique, postoperative quality of life, and long-term effects.

### Technique

- The implant gravity effect is counteracted by the muscle closure—an inherent problem to all existing techniques.
- Any size implant can be used provided that there is complete muscle coverage. Our implant size range has been 150-375 cc (μ = 270 cc).
- We always used round silicone smooth implants of moderate or moderate plus profile. Saline implants can also be used. Smooth implants are not palpable and have movement within the muscle pocket. Smooth implants are also preferred due to the current anaplastic large cell lymphoma issue.
- No muscle animation, upper pole ridging, or medial and lateral rippling are seen. The pectoralis major muscle is sutured and stabilized inferiorly to the muscle cuff and remains stretched. This stretching of the pectoralis major creates a tenting effect over the implant. The wide undermining of the inferior pedicle allows the breast tissue to be freely redistributed superiorly, anterior to the implant, thus creating an additional layer of tissue to make the implant almost unnoticeable and not palpable. Importantly, the pinch test is not a guiding indicator for our procedure.
- The implants never extrude due to the muscle coverage. Even in cases of “T” sloughing or skin flap necrosis, the implants will never be exposed.
- Due to the muscle support, there is no loss of cleavage, implant malpositioning, and bottoming even after pregnancies and lactation.
- The inferior pedicle mastopexy and the absence of the periareolar approach for the augmentation leave the breast tissue intact for lactation. Therefore, there is no ductal obstruction and lactation is uneventful.
- Any degree of breast ptosis, including bariatric cases, can be corrected with this 1-stage technique. There is no vascular compromise to the inferior pedicle with the procedure, and, therefore, there is no limitation of the length of the pedicle, the length of the vertical excess, and the nipple elevation.
- This technique provides a tight skin envelope over the redistributed parenchyma, due to the wide undermining, and makes the result long lasting.
- The plication of the inferior pedicle provides support to the inferior pedicle and reduces the degree of breast tissue bottoming. It is also an easy way to correct breast asymmetry.
- No need for staging. No need for early-stage reoperations for corrective purposes as reported in 1-stage augmentation-mastopexies using other techniques.
- Accellular Dermal Matrix (ADM) are not used. The presence of muscular support and capsular tissue makes ADMs unnecessary for this procedure and for any long-term revisions.

### Postoperative Quality of Life

- The stabilization of the pectoralis muscle decreases the postoperative pain. The patient is able to fully extend their arms without pain in 3 days and requires minimal opioids for pain management for the first 24 hours.
- We believe that this is due to the stabilization of the pectoralis muscle to the muscle cuff.
- There is no need for the use of a postoperative bra because an internal muscular bra has been constructed. The patient may choose to wear a soft bra or a sports bra electively for comfort for as long as they prefer. However, the postoperative result is not affected by the use of the bra.
- With the true submuscular placement, the smooth implants are not palpable, and the patients can barely feel their presence. Palpability is a parameter that is hardly discussed in the literature of breast surgery with implants. The implants that are palpable after the augmentation and “dance under the skin” at the inferior pole in the upright position is an issue that is not addressed and leaves the patients dissatisfied.

### Long-term Effects

Long-term effects such as the naturally aging breast, capsular contracture, high double bubble, waterfall phenomenon, or bottoming of breast tissue due to poor quality of the skin, especially in bariatric cases, can be safely corrected by always maintaining the true submuscular pocket and combining any of the following modalities. It is noteworthy that the combination of the following modalities does not run the risk of vascular compromise or extrusion of the implants, as long as the muscle pocket is respected.

- Anterior and peripheral capsulotomy/partial capsulectomy/ capsulorrhaphy.
- Change of implant size: The submuscular pocket stretches and the size of the implants can be increased easily an average of 100-125 cc and sometimes more, after a 4- to 6-month period, maintaining the muscle pocket intact. Smaller implants can be used in cases of excessive weight gain.
- Permanent explantation with crescentic excision of the lower pole if the nipples are in a proper place, or explantation with redo of the inferior pedicle in the unlikely event the position of the nipples needs to be revised.
- Lowering of IMF by deepening the muscle cuff in order to improve small lower pole ptosis due to aging and sagging breast.
- Crescentic excision of the lower pole without vascular compromise.
- No need for 2-stage revisions or staging a temporary explantation. ADMs are not required for any revisional surgery of this procedure.

## Conclusions

The major benefits of this technique for a long-lasting result are derived from the complete muscular coverage of the implants and the versatility of the tightening of the skin envelope that the inferior pedicle allows. The procedure makes it also technically easy, without the use of ADMs, to perform any future maintenance procedures of the aging breast without running the risk of implant bottoming, malpositioning, extrusion, or tissue necrosis.

We strongly believe that this is a breakthrough approach to the 1-stage augmentation mastopexy procedure for primary grades 2 and 3 ptosis of any size including bariatric breast ptosis. The prerequisite for the split inferior pedicle mastopexy is the use of the TESMA for the augmentation. It has been proven in our practice to be a safe, simple, and reproducible procedure that is easy to be taught and very reliable without any major complications.
